# Qualitative and quantitative comparison of TGRAPPA and TSENSE real-time cine techniques during deep breathing

**DOI:** 10.1186/1532-429X-11-S1-P217

**Published:** 2009-01-28

**Authors:** Mihaela Jekic, Yu Ding, Jennifer Dickerson, Ali Merchant, YiuCho Chung, Orlando P Simonetti

**Affiliations:** 1 Ohio State University, Columbus, OH USA; 2 Siemens Medical Solutions, Columbus, OH USA

**Keywords:** Temporal Resolution, Deep Breathing, Artifact Severity, Superior Image Quality, Rapid Heart Rate

## Objetive

To compare the performance of real-time cine imaging using TSENSE and TGRAPPA acceleration methods during deep breathing.

## Background

The rapid heart rates and heavy breathing encountered during exercise stress MRI make it difficult to achieve the necessary spatial resolution, temporal resolution, and image quality with real-time cine techniques. Parallel imaging has enabled improved temporal resolution, but at the cost of increased artifact and noise. Temporal resolution can be further improved by reducing FOV in the phase encoded direction. However, in the case of deep breathing during or after exercise, the chest wall can move in and out of the FOV causing aliasing. Surface coils can also move with deep breathing, causing a mismatch between the coil sensitivity map and the actual coil position, another potential source of artifact [1]. GRAPPA is known to be less sensitive than SENSE to these effects [2]. TSENSE [3] and TGRAPPA [1] are techniques for dynamic imaging that derive coil sensitivity maps by interleaving and averaging the undersampled dynamic frames. Our hypothesis is that TGRAPPA will perform better than TSENSE under the condition of deep breathing that causes aliasing and coil sensitivity map errors.

## Methods

We acquired cine series in three views (short-axis, vertical and horizontal long-axis) in 5 healthy subjects during deep breathing using SSFP real-time cine accelerated 3-fold with TSENSE and TGRAPPA on a Siemens 1.5 T Avanto, resulting in a total of 30 image series. Sensitivity maps were obtained by interleaving and averaging all undersampled frames in each series. Scan parameters were: TE/TR 0.9/2.2 ms, average FOV 374 × 299mm, matrix 160 × 84, and temporal resolution 63.8 ± 1.6 ms. The FOV was specified immediately adjacent to the chest wall in the view in which the body appears largest in the phase encode direction (typically the VLA). Two experienced readers blinded to the study assigned qualitative scores of artifact severity: (1) none, (2) minor, (3) moderate, and (4) severe, and overall image quality: (1) excellent, (2) good, (3) diagnosis may be limited, and (4) poor, non-diagnostic. Artifacts were also quantitatively analyzed by examining peaks in the image autocorrelation function in the phase encode direction at shifts of 1/3 and 2/3 FOV, the expected locations for parallel imaging associated ghost artifacts at rate 3 acceleration. Within each series, the highest 10% of autocorrelation coefficients at 1/3 FOV were averaged to define an "artifact index".

## Results

**Figure 1:**
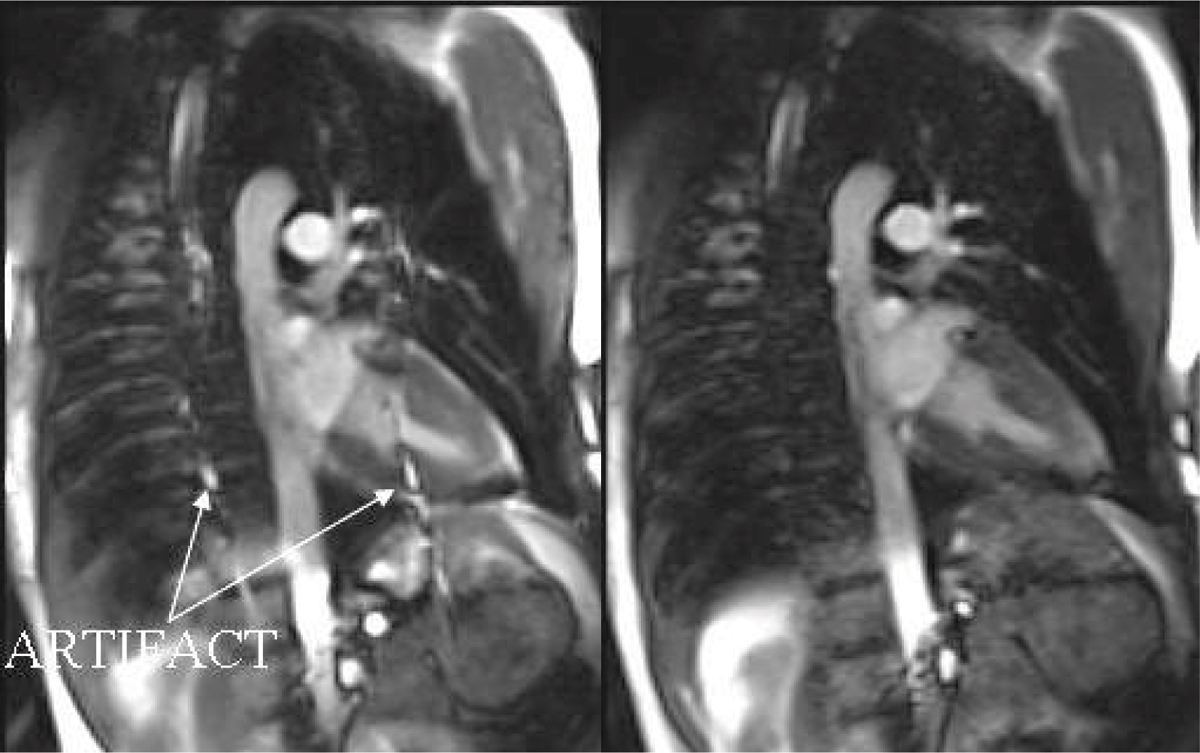
**Increased artifact severity with TSENSE** ***(left)*** **vs. TGRAPPA** ***(right)*** **under deep breathing**.

TGRAPPA scores were superior to TSENSE both in terms of the physician-assessed image quality (1.6 ± 0.6 vs. 2.3 ± 0.7, p < .001) and artifact severity (1.7 ± 0.6 vs. 2.9 ± 0.8, p < .001). The computed artifact index agreed with the qualitative artifact score, with TGRAPPA showing less artifact severity (0.058 ± 0.035 vs. 0.110 ± 0.052, p = .0036). Figure 1 illustrates the increased artifact level with TSENSE compared to TGRAPPA.

## Conclusion

In the presence of deep breathing, TGRAPPA delivered superior image quality and artifact performance compared to TSENSE independent of FOV and aliasing, and exhibited less sensitivity to coil map errors. While SENSE provides an optimal reconstruction under normal conditions of breath-hold or quiet breathing, it is known to suffer from artifacts when the FOV is smaller than the object or the coil map is imperfect [2]. Our results indicate that TGRAPPA is preferred over TSENSE under conditions such as exercise stress, deep breathing, and patient motion.
